# Association of genetic variants in lncRNA *H19* with risk of colorectal cancer in a Chinese population

**DOI:** 10.18632/oncotarget.8330

**Published:** 2016-03-24

**Authors:** Shuwei Li, Yibing Hua, Jing Jin, Haixiao Wang, Mulong Du, Lingjun Zhu, Haiyan Chu, Zhengdong Zhang, Meilin Wang

**Affiliations:** ^1^ Department of Environmental Genomics, Jiangsu Key Laboratory of Cancer Biomarkers, Prevention and Treatment, Collaborative Innovation Center for Cancer Personalized Medicine, Nanjing Medical University, Nanjing, China; ^2^ Department of Genetic Toxicology, The Key Laboratory of Modern Toxicology of Ministry of Education, School of Public Health, Nanjing Medical University, Nanjing, China; ^3^ Department of General Surgery, The First Affiliated Hospital of Nanjing Medical University, Nanjing, China; ^4^ Department of General Surgery, Huai-An First People's Hospital Affiliated to Nanjing Medical University, Huai-an, China; ^5^ Department of Oncology, The First Affiliated Hospital of Nanjing Medical University, Nanjing, China

**Keywords:** long noncoding RNA, H19, colorectal cancer, genetic variation, susceptibility

## Abstract

**Objective:**

The long non-coding RNA (lncRNA) gene, *H19*, has been involving in multiple biological functions, which also plays a vital role in colorectal cancer carcinogenesis. However, the association between genetic variants in *H19* and colorectal cancer susceptibility has not been reported. In this study, we aim to explore whether *H19* polymorphisms are related to the susceptibility of colorectal cancer.

**Methods:**

We conducted a case-control study to evaluate the association between four selected single nucleotide polymorphisms (SNPs) (rs2839698, rs3024270, rs217727, and rs2735971) in *H19* and the risk of colorectal cancer in a Chinese population.

**Results:**

We found that individuals with rs2839698 A allele had a significantly increased risk of colorectal cancer, compared to those carrying G allele [odds ratio (OR) = 1.20, 95% confidence interval (CI) = 1.05–1.36 in additive model]. Further stratified analyses revealed that colon tumor site, well differentiated grade and Duke's stage of C/D were significantly associated with colorectal cancer risk (*P* < 0.05). Additionally, bioinformatic analysis showed that rs2839698 may change the crucial folding structures and alter the target microRNAs of *H19*.

**Conclusions:**

Our results provided the evidence that rs2839698 in *H19* was associated with elevated risk of colorectal cancer, which may be a potential biomarker for predicting colorectal cancer susceptibility.

## INTRODUCTION

Colorectal cancer is the most-common malignant tumor worldwide, with over 132,700 new cases and 49,700 deaths estimated every year in the United State [1]. Epidemiological data reported by the International Agency for Research on Cancer (IARC) demonstrated that colorectal cancer accounts for 8.3% and 6.3% of all malignancies incidence and mortality in China, respectively [2]. The occurrence and development of colorectal cancer is caused by a series of multifactorial and complex factors including environmental alterations and genetic aspects [3, 4]. In recent years, considerable genome-wide association studies (GWAS) have identified numerous genetic variants impacting the risk of colorectal cancer [5–8]. Zhang *et al*. conducted a GWAS in East Asians and identified 6 new loci associated with colorectal cancer risk [9]. In addition, Jia *et al*. identified three new colorectal cancer susceptibility loci [10]. These studies provide additional insights into the genetic and biological basis of colorectal cancer.

As we known, the abnormality of gene expression may increase the risk or severity of diseases [11–13]. Long noncoding RNAs (lncRNAs) have also been implicated in the crucial functions of various biological process involved in cancer susceptibility [14, 15]. The lncRNA *H19*, highly conserved on chromosome 11p15.5 in human, is a maternal expressed gene that plays key roles in embryogenesis during fetal time [16, 17]. However, it is down-expressed in maturing tissues postnatal [18]. Accumulating evidences suggested that *H19* was up-regulated in a variety of cancer types, including breast cancer [19, 20], esophageal cancer [21], bladder cancer [22] and colorectal cancer [23]. In addition, the differentially methylated regions (DMRs), which located upstream of the transcription start of *H19*, act the part of methylation-sensitive insulator [24]. Furthermore, emerging studies indicated that *H1*9 may activate tumorigenicity by acting as the precursors of microRNAs (miRNAs) or competitive endogenous RNAs (ceRNAs) [25–27]. Tsang *et al*. observed that miR-675, derived from *H19*, may decrease the expression of retinoblastoma (*RB*) and increase the growth and development of colorectal cancer cells [28]. Induction of epithelial-mesenchymal transition (EMT) in cancer cells due to aberrant *H19* expression can promote pancreatic ductal adenocarcinoma cell invasion and migration [29].

Recently several single nucleotide polymorphisms (SNPs) within lncRNA genes have been extensively confirmed to modulate the expression and function of lncRNA and further cause tumor susceptibility and prognosis changing [30, 31]. As for SNPs in lncRNA *H19*, cumulative studies have identified the associated with malignant diseases [32, 33]. In this study, we conducted a case-control study to genotype the candidate SNPs in *H19* (rs2839698, rs3024270, rs217727, and rs2735971) and investigate the association with the risk of colorectal cancer.

## RESULTS

### Characteristics of the study subjects

1147 colorectal cancer patients and 1203 controls were recruited in this study. No significant differences were observed regarding to age and gender between patients and cancer-free controls (*P* = 0.751 and *P* = 0.116, respectively), indicating satisfactory matching by these factors. There were no significant differences in smoking and drinking status between the patients and controls (*P* > 0.05). However, more colorectal cancer individuals were found to have family history of cancers than subjects in control (*P* < 0.001). For tumor grade, 7.4% of colorectal cancer cases were in low grade, and 76.7% in the intermediate, and 15.9% in the high grade. Moreover, the frequencies of the tumor Duke's stage were 8.4% (A), 43.1% (B), 36.8% (C) and 11.7% (D).

### Associations of selected SNPs in *H19* and colorectal cancer risk

**Figure 1:**
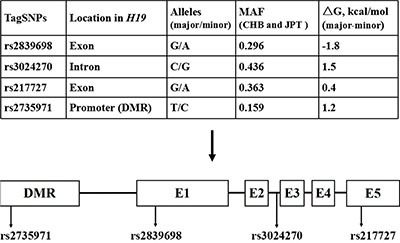
Selected tagSNPs and relative position in *H19* E1, E2, E3, E4, E5 and DMR indicates exon 1, exon 2, exon 3, exon 4, exon 5, and differentially methylated regions, respectively, whereas the line indicates the introns.

The positions of four selected SNPs in *H19* are shown in Figure 1. Primary information and the distributions of genotypes were consistent with those expected from Hardy-Weinberg equilibrium (HWE) in the control group (*P* = 0.666 for rs2839698, *P* = 0.979 for rs3024270, *P* = 0.959 for rs217727 and *P* = 0.175 for rs2735971, respectively. Besides, we calculated genotype frequencies of *H19* tagSNPs among cases and controls and their associations with colorectal cancer risk according to variant genetic effect models (additive, dominant, recessive and co-dominant models) (Table 1, Supplementary Table S1). As a result, we observed that rs2839698 was significantly associated with the risk of colorectal cancer after the adjustment for age, gender, smoking and drinking status by performing multivariate logistic regression analysis in additive model [odds ratios (ORs) = 1.20, 95% confidence intervals (CIs) = 1.05–1.36, *P* = 0.007 and *P* = 0.028 after Bonferroni correction]. No significant association between rs3024270, rs217727 and rs2735971 and colorectal cancer risk were found in the additive model.

**Table 1:** Associaiton between the selected tagSNPs and the risk of colorectal cancer

SNPs	Location^a^	Position	Alleles^b^	Cases^c^(n = 1147)	Controls^c^(n = 1203)	MAF^d^(Case/control)	*P*_HWE_^e^	Adjusted OR (95% CI)^f^					*P*^h^	*P*^i^
								Additive model	Dominant model	Recessive model	Codominant model^g^			
											het	hom		
rs2839698	2018853	Exon1	G/A	583/462/102	666/462/75	0.290/0.254	0.666	**1.20 (1.05-1.36)**	**1.20 (1.02-1.41)**	**1.46 (1.07-1.99)**	1.14 (0.96-1.35)	**1.54 (1.12-2.12)**	0.007	0.028
rs3024270	2017439	Intron	C/G	385/527/235	420/582/201	0.435/0.409	0.979	1.11 (0.99-1.24)	1.06 (0.90-1.26)	**1.29 (1.04-1.58)**	0.99 (0.83-1.19)	**1.28 (1.01-1.61)**	0.079	0.316
rs217727	2016908	Exon5	G/A	480/514/153	456/570/177	0.357/0.334	0.959	0.89 (0.79-1.00)	0.84 (0.71-1.00)	0.89 (0.71-1.13)	0.85 (0.71-1.01)	0.82 (0.64-1.06)	0.056	0.224
rs2735971	2021649	Promoter (DMR)	T/C	773/334/40	765/398/40	0.180/0.199	0.175	0.89 (0.77-1.03)	0.85 (0.72-1.01)	1.06 (0.68-1.66)	0.83 (0.70-1.00)	1.00 (0.64-1.57)	0.125	0.500

Abbreviations: OR, odds ratio; Cl, confidence interval.

a Location in GRCh 37.

b Major/minor.

c Numbers of major homozygote/heterozygote/minor homozygote.

d Minor allele frequency in cases/controls.

e HWE, Hardy-Weinberg equilibrium in control subjects.

f Adjusted for age, sex, smoking and drinking status in logistic regression model.

g het: heterozygote versus major homozygote; hom: minor homozygote versus major homozygote.

h *P* for additive model.

i *P* after Bonferroni correction.

### Stratification analysis of associations between rs2839698 and colorectal cancer

To exclude whether the possible confounders play roles in the colorectal cancer risk, we conducted the stratified analysis upon the associations between rs2839698 and colorectal cancer by age, sex, smokers, drinkers and family history of cancers. Due to the small number of AA genotype group, we performed the stratification analysis under dominant model. As shown in Table 2, more profoundly increased risk of colorectal cancer were identified in terms of younger subjects (age ≤ 61) (*P* = 0.007), males (*P* = 0.022), drinkers (*P* = 0.008) and smokers (*P* = 0.002).

**Table 2:** Stratification analyses for rs2839698 genotypes and colorectal cancer risk

Variables	Genotypes for rs2839698 (cases/controls)				Adjusted OR (95% CI)	*P*^a^
	GA/AA		GG			
	N	%	N	%		
Age (years)						
≤ 61	303/251	50.2/42.7	300/337	49.8/57.3	**1.39 (1.10-1.76)**	0.007
> 61	261/286	48.0/46.5	283/329	52.0/53.5	1.07 (0.84-1.37)	0.560
Sex						
Male	349/306	49.7/43.8	353/392	50.3/56.2	**1.29 (1.04-1.61)**	0.022
Female	215/231	48.3/45.7	230/274	51.7/54.3	1.15 (0.88-1.50)	0.296
Smoking status						
Never	344/369	46.8/45.5	392/442	53.3/54.5	1.05 (0.86-1.29)	0.635
Ever	220/168	53.5/42.9	191/224	46.5/57.1	**1.57 (1.18-2.10)**	0.002
Drinking status						
Never	393/408	47.8/45.4	430/490	52.2/54.6	1.11 (0.92-1.35)	0.276
Ever	171/129	52.8/42.3	153/176	47.2/57.7	**1.58 (1.13-2.20)**	0.008
FH						
No	444/477	49.1/44.3	460/599	50.9/55.7	**1.21 (1.01-1.45)**	0.042
Yes	120/60	49.4/47.2	123/67	50.6/52.8	1.20 (0.77-1.89)	0.422

a Adjusted for age, sex, smoking and drinking status in logistic regression models. Abbreviations: OR, odds ratio; CI, confidence interval; FH, family history of cancers.

### Association between rs2839698 and clinicopathologic characteristics of colorectal cancer

Next, we performed the subgroup analysis in different clinicopathologic variables to evaluate the relationship between rs2839698 and colorectal cancer risk. As shown in Supplementary Table S2, rs2839698 GA/AA genotypes were associated with an increased risk of colorectal cancer in individuals with colon tumor site (OR = 1.25, 95% CI = 1.02–1.52, *P* = 0.033), well differentiated grade (OR = 1.54, 95% CI = 1.13–2.11, *P* = 0.007) and Duke's C/D stage (OR = 1.37 95% CI = 1.12–1.68, *P* = 0.002). However, no dramatically significant risk effect of colorectal cancer was observed in other subgroups.

### Prediction of rs2839698 on *H19* folding structures and target miRNAs

**Figure 2:**
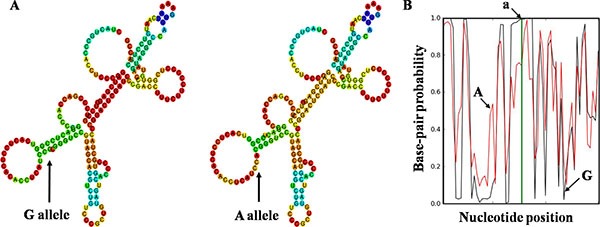
Bioinformatics prediction of rs2839698 on *H19* folding structure The folding structure alterations were demonstrated by RNAfold (**A**) and SNPfold (**B**), respectively. Arrow (A) indicates the change in structure caused by rs2839698. Arrow a indicates the position of rs2839698. Arrow G indicates the sequences of G allele, whereas arrow A indicates the A allele.

We performed *in silico* analyses using RNAfold and SNPfold to predict the *H19* secondary structure of selected SNPs. As a result, the secondary structure was dramatically changed with rs2839698 G/A alleles (Figure 2), rs3024270 C/G alleles and rs217727 G/A alleles (Supplementary Figure S1). However, there were few changes with rs2735971 T/C alleles.

According to the fact that rs2839698 (G/A) is located in the exon (3′untranslated region) of *H19* gene, we speculate that genetic variant in rs2839698 may change the promoter activity and function of *H19* to a certain extent through alteration of target miRNAs and subsequently lead to colorectal cancer. Consequently, we used miRNASNP v2.0 to predict whether rs2839689 (G/A) in 3′untranslated region of *H19* gene can induce target miRNAs gain/loss. We found that hsa-miR-24–1–5p, hsa-miR-4486, hsa-miR-566 and hsa-miR-24–2–5p may lose the target *H19* gene, following with the creating binding site of hsa-miR-612, hsa-miR-5189, hsa-miR-1285–3p and hsa-miR-3187–5p (Supplementary Table S3).

## DISCUSSION

*H*19 is a paternally imprinted oncofetal lncRNA gene locus on chromosome 11p15.5 which is down-regulated after birth and possesses oncogenic properties. Previous studies have indicated that *H19* involves in the complex biological process of oncogenesis [26, 34, 35]. Liang *et al*. reported that the lncRNA *H19* play the part of miRNA sponges to promoting EMT in colorectal cancer [36]. *H19* may also act as a primary miRNA precursor to continue the function [37]. Moreover, *H19* has the potential to produce *91H* RNA, which regulates insulin like growth factor 2 (*IGF2*) expression and is over-expressed in breast cancer cells [38]. Despite of extensive evidence, the function of *H19* in the molecular mechanism of tumorigenesis is still not clear. Emerging evidences have implied that genetic variants in lncRNAs may modify the risk of multiply tumors [30, 39]. Verhaegh *et al*. have found that *H19* gene polymorphisms were concerned in susceptibility of bladder cancer in European Caucasians [33]. However, to our knowledge, no data previously has explored the correlation between *H19* genetic variants and colorectal cancer susceptibility in a Chinese population.

In this study, we selected four tagSNPs (rs2839698, rs3024270, rs217727, and rs2735971) in *H19* gene and DMR to estimate the association between these variants and colorectal cancer susceptibility. We observed that rs2839698 GA/AA genotype has an increased risk of colorectal cancer in the Chinese populations compared with the GG genotype. Yang *et al*. also demonstrated that rs2839698 contributed to the risk of gastric cancer in a Chinese population [32]. All of the above suggested that *H19* genetic variants play an important role in cancer susceptibility.

Some environmental factors, such as alcohol intake and tobacco smoking, were related with the elevated colorectal cancer risk [40–42]. Our stratified analyses demonstrated that individuals, including smokers and drinkers, carrying rs2839698 GA/AA genotype had a significantly increased susceptibility of colorectal cancer. Therefore, the markedly induced risk of colorectal cancer associated with variant rs2839698 genotypes could partly attribute to the accumulated exposure/exposure history to alcohol consumption or tobacco carcinogens. Moreover, we found that increased colorectal cancer risk correlated with rs2839698 was more remarkable in subgroups of younger individuals and male, suggested that promoting effects of *H19* variants on colorectal cancer may be modulated by specific epidemiological features. These results provided confirmed that colorectal cancer tumorigenesis is a complex and multistep process involving diverse genetic and environmental modifications. However, we found that the associations of colorectal cancer risk in subjects with and without family history of cancers were almost the same. It is reasonable that the number of controls with family history was less than the patients.

We further found that rs2839698 GA/AA genotype had an increased risk of colorectal cancer among patients with Duke's stage of C or D. It is rational that the genetic variants may play a vital role in the advanced stage of colorectal cancer and lead to our present result. However, the results showed that subjects with the gene loci variation were involved in the obviously increased risk of colorectal cancer among colon site and well differentiated grade subgroups, indicating that different colorectal cancer site and grade regulated by different molecular biological mechanisms may bring about different level of risk in carcinogenesis of colorectal cancer [43].

Given the important function influence of folding structure changes of lncRNAs caused by SNPs, we predicted the secondary structure changes of *H19* ascribed to selected SNPs using RNAfold and SNPfold algorithms. We found that the folding architectures markedly changed along with the genetic variant of rs2839689, rs3024270, rs217727, and rs2735971, suggested that SNPs may be involved in occurrence and development of colorectal cancer by altering the specific structural motifs of *H19* and exerting various effects on *H19* expression and function [44]. Besides, accumulating studies have revealed that SNPs in lncRNAs can be directly regulated and modified by miRNAs [34, 43], and SNPs might be plausible reason for alteration of interactions between miRNAs and lncRNAs [45]. Based on previous evidence, miRNASNP v2.0 was used to predict the lost miRNAs of wild sequence and the obtained miRNAs of SNP sequence. We found that four obtained miRNAs and four lost miRNAs possibly linked with lncRNA *H19*. The changes of target miRNAs may potentially affect the expression and function of *H19* due to rs2839689 variant, which ultimately modulate the risk of colorectal cancer.

In summary, we have provided the evidence that *H19* rs2839689 contributes to the susceptible to colorectal cancer in the Chinese population. Further both larger prospective studies and functional researches are needed to validate the finding in different ethnicities.

## MATERIALS AND METHODS

### Study participants

The present study was approved by the Institutional Review Board of Nanjing Medical University. All the study participants were genetically unrelated Chinese and provided written informed consent. Briefly we consecutively recruited 1,147 patients with colorectal cancer and 1,203 cancer-free controls. All cases were histopathologically confirmed colorectal tumor from the Affiliated Nanjing First Hospital and the First Affiliated Hospital of Nanjing Medical University on September 2010, without age or sex restrictions. Control individuals were matched to the cases based upon age (± 5 years) and sex. Details of the study participants have been demonstrated previously [39, 46].

### SNP selection

We focused on both lncRNA *H19* gene and its promoter (including DMR) located in human chromosome 11p15.5 using UCSC browser (http://genome.ucsc.edu/). Four SNPs were selected on the basis of four filtering criteria: (a) minor allele frequency (MAF) > 0.05 in the CHB and JPT population from the 1000 Genomes Project; (b) *r*^2^ > 0.8 analyzed based on pairwise linkage disequilibrium using Haploview version 4.0; (c) the secondary structure changed using RNAfold; (d) the Gibbs binding free energy (ΔG, kJ/mol) > 0.

### Genotyping

Genotyping was performed using the TaqMan allelic discrimination assay. The 384-well ABI 7900HT real-time PCR system (Applied Biosystems, Foster City, CA, USA) was applied to amplify all the sample genotypes, with SDS 2.4 software (Applied Biosystems) used to read and analyze allelic discrimination. Both the sequences of the primers and fluorescent probes are showed in Supplementary Table S4. The average call rates for four SNPs were more than 99%. Additionally, we randomly selected over 10% of the samples for repeated assays and the final concordance rate between duplicate samples was 100%.

### *In silico* prediction of secondary structures and target miRNAs

We used RNAfold (http://rna.tbi.univie.ac.at/) [47] and SNPfold (http://ribosnitch.bio.unc.edu/snpfold/SNPfold.html) [44] algorithms to predict the folding structure variants of *H19* on account of tagSNPs genotypes. In addition, miRNASNP v2.0 (http://bioinfo.life.hust.edu.cn/miRNASNP2/) was used to predict the target miRNAs of *H19*.

### Statistical analysis

Differences in the distribution of epidemiological variables between cases and controls were calculated using Student's *t*-tests (continuous variables) and chi-square χ^2^ tests (categorical variables). The crude and adjusted ORs and 95% CIs were using to examine the correlation between different genotypes and colorectal cancer risk from unconditional univariate as well as multivariate logistic regression analyses under variant genetic models. Age, sex and smoking and drinking status were involved in the possible confounders in order to perform multivariate logistic regression analyses. HWE in the cancer-free groups was computed by a goodness-of fit chi-square test. Linkage equilibrium (LD) between SNPs in *H19* was calculated using Haploview 4.0 software. Bonferroni correction was applied to conservatively account for multiple comparisons. All outputted *P*-values were 2-sided and the criterion of *P*-value for statistical significance is less than 0.05. Moreover, all of the tests were performed using SAS software package (version 9.1.3; SAS Institute, Inc., Cary, NC).

## SUPPLEMENTARY MATERIALS FIGURE AND TABLES



## References

[1] Siegel RL, Miller KD, Jemal A (2015). Cancer statistics, 2015. CA Cancer J Clin.

[2] Ferlay J, Soerjomataram I, Dikshit R, Eser S, Mathers C, Rebelo M, Parkin DM, Forman D, Bray F (2015). Cancer incidence and mortality worldwide: sources, methods and major patterns in GLOBOCAN 2012. Int J Cancer.

[3] Haggar FA, Boushey RP (2009). Colorectal cancer epidemiology: incidence, mortality, survival, and risk factors. Clin Colon Rectal Surg.

[4] Lichtenstein P, Holm NV, Verkasalo PK, Iliadou A, Kaprio J, Koskenvuo M, Pukkala E, Skytthe A, Hemminki K (2000). Environmental and heritable factors in the causation of cancer—analyses of cohorts of twins from Sweden, Denmark, and Finland. N Engl J Med.

[5] Tenesa A, Dunlop MG (2009). New insights into the aetiology of colorectal cancer from genome-wide association studies. Nat Rev Genet.

[6] Cui R, Okada Y, Jang SG, Ku JL, Park JG, Kamatani Y, Hosono N, Tsunoda T, Kumar V, Tanikawa C, Kamatani N, Yamada R, Kubo M (2011). Common variant in 6q26-q27 is associated with distal colon cancer in an Asian population. Gut.

[7] Theodoratou E, Montazeri Z, Hawken S, Allum GC, Gong J, Tait V, Kirac I, Tazari M, Farrington SM, Demarsh A, Zgaga L, Landry D, Benson HE (2012). Systematic meta-analyses and field synopsis of genetic association studies in colorectal cancer. J Natl Cancer Inst.

[8] Schumacher FR, Schmit SL, Jiao S, Edlund CK, Wang H, Zhang B, Hsu L, Huang SC, Fischer CP, Harju JF, Idos GE, Lejbkowicz F, Manion FJ (2015). Genome-wide association study of colorectal cancer identifies six new susceptibility loci. Nat Commun.

[9] Zhang B, Jia WH, Matsuda K, Kweon SS, Matsuo K, Xiang YB, Shin A, Jee SH, Kim DH, Cai Q, Long J, Shi J, Wen W (2014). Large-scale genetic study in East Asians identifies six new loci associated with colorectal cancer risk. Nat Genet.

[10] Jia WH, Zhang B, Matsuo K, Shin A, Xiang YB, Jee SH, Kim DH, Ren Z, Cai Q, Long J, Shi J, Wen W, Yang G (2013). Genome-wide association analyses in East Asians identify new susceptibility loci for colorectal cancer. Nat Genet.

[11] Anstee QM, Seth D, Day CP (2016). Genetic Factors That Affect Risk of Alcoholic and Non-Alcoholic Fatty Liver Disease. Gastroenterology.

[12] Hoffmann C, Mao X, Dieterle M, Moreau F, Al Absi A, Steinmetz A, Oudin A, Berchem G, Janji B, Thomas C (2016). CRP2, a new invadopodia actin bundling factor critically promotes breast cancer cell invasion and metastasis. Oncotarget.

[13] Ke X, Li Q, Xu L, Zhang Y, Li D, Ma J, Mao X (2015). Netrin-1 overexpression in bone marrow mesenchymal stem cells promotes functional recovery in a rat model of peripheral nerve injury. J Biomed Res.

[14] Gupta RA, Shah N, Wang KC, Kim J, Horlings HM, Wong DJ, Tsai MC, Hung T, Argani P, Rinn JL, Wang Y, Brzoska P, Kong B (2010). Long non-coding RNA HOTAIR reprograms chromatin state to promote cancer metastasis. Nature.

[15] Wang KC, Chang HY (2011). Molecular mechanisms of long noncoding RNAs. Mol Cell.

[16] Gabory A, Ripoche MA, Yoshimizu T, Dandolo L (2006). The H19 gene: regulation and function of a non-coding RNA. Cytogenet Genome Res.

[17] Gabory A, Jammes H, Dandolo L (2010). The H19 locus: role of an imprinted non-coding RNA in growth and development. Bioessays.

[18] Thorvaldsen JL, Duran KL, Bartolomei MS (1998). Deletion of the H19 differentially methylated domain results in loss of imprinted expression of H19 and Igf2. Genes Dev.

[19] Adriaenssens E, Dumont L, Lottin S, Bolle D, Lepretre A, Delobelle A, Bouali F, Dugimont T, Coll J, Curgy JJ (1998). H19 overexpression in breast adenocarcinoma stromal cells is associated with tumor values and steroid receptor status but independent of p53 and Ki-67 expression. Am J Pathol.

[20] Berteaux N, Lottin S, Monte D, Pinte S, Quatannens B, Coll J, Hondermarck H, Curgy JJ, Dugimont T, Adriaenssens E (2005). H19 mRNA-like noncoding RNA promotes breast cancer cell proliferation through positive control by E2F1. J Biol Chem.

[21] Hibi K, Nakamura H, Hirai A, Fujikake Y, Kasai Y, Akiyama S, Ito K, Takagi H (1996). Loss of H19 imprinting in esophageal cancer. Cancer Res.

[22] Luo M, Li Z, Wang W, Zeng Y, Liu Z, Qiu J (2013). Long non-coding RNA H19 increases bladder cancer metastasis by associating with EZH2 and inhibiting E-cadherin expression. Cancer Lett.

[23] Cui H, Onyango P, Brandenburg S, Wu Y, Hsieh CL, Feinberg AP (2002). Loss of imprinting in colorectal cancer linked to hypomethylation of H19 and IGF2. Cancer Res.

[24] Gao T, He B, Pan Y, Gu L, Chen L, Nie Z, Xu Y, Li R, Wang S (2014). H19 DMR methylation correlates to the progression of esophageal squamous cell carcinoma through IGF2 imprinting pathway. Clin Transl Oncol.

[25] Keniry A, Oxley D, Monnier P, Kyba M, Dandolo L, Smits G, Reik W (2012). The H19 lincRNA is a developmental reservoir of miR-675 that suppresses growth and Igf1r. Nat Cell Biol.

[26] Matouk IJ, Halle D, Raveh E, Gilon M, Sorin V, Hochberg A (2016). The role of the oncofetal H19 lncRNA in tumor metastasis: orchestrating the EMT-MET decision. Oncotarget.

[27] Xia T, Liao Q, Jiang X, Shao Y, Xiao B, Xi Y, Guo J (2014). Long noncoding RNA associated-competing endogenous RNAs in gastric cancer. Sci Rep.

[28] Tsang WP, Ng EK, Ng SS, Jin H, Yu J, Sung JJ, Kwok TT (2010). Oncofetal H19-derived miR-675 regulates tumor suppressor RB in human colorectal cancer. Carcinogenesis.

[29] Ma C, Nong K, Zhu H, Wang W, Huang X, Yuan Z, Ai K (2014). H19 promotes pancreatic cancer metastasis by derepressing let-7′s suppression on its target HMGA2-mediated EMT. Tumour Biol.

[30] Du M, Wang W, Jin H, Wang Q, Ge Y, Lu J, Ma G, Chu H, Tong N, Zhu H, Wang M, Qiang F, Zhang Z (2015). The association analysis of lncRNA HOTAIR genetic variants and gastric cancer risk in a Chinese population. Oncotarget.

[31] Tao R, Hu S, Wang S, Zhou X, Zhang Q, Wang C, Zhao X, Zhou W, Zhang S, Li C, Zhao H, He Y, Zhu S (2015). Association between indel polymorphism in the promoter region of lncRNA GAS5 and the risk of hepatocellular carcinoma. Carcinogenesis.

[32] Yang C, Tang R, Ma X, Wang Y, Luo D, Xu Z, Zhu Y, Yang L (2015). Tag SNPs in long non-coding RNA H19 contribute to susceptibility to gastric cancer in the Chinese Han population. Oncotarget.

[33] Verhaegh GW, Verkleij L, Vermeulen SH, den Heijer M, Witjes JA, Kiemeney LA (2008). Polymorphisms in the H19 gene and the risk of bladder cancer. Eur Urol.

[34] Kallen AN, Zhou XB, Xu J, Qiao C, Ma J, Yan L, Lu L, Liu C, Yi JS, Zhang H, Min W, Bennett AM, Gregory RI (2013). The imprinted H19 lncRNA antagonizes let-7 microRNAs. Mol Cell.

[35] Raveh E, Matouk IJ, Gilon M, Hochberg A (2015). The H19 Long non-coding RNA in cancer initiation, progression and metastasis - a proposed unifying theory. Mol Cancer.

[36] Liang WC, Fu WM, Wong CW, Wang Y, Wang WM, Hu GX, Zhang L, Xiao LJ, Wan DC, Zhang JF, Waye MM (2015). The lncRNA H19 promotes epithelial to mesenchymal transition by functioning as miRNA sponges in colorectal cancer. Oncotarget.

[37] Cai X, Cullen BR (2007). The imprinted H19 noncoding RNA is a primary microRNA precursor. RNA.

[38] Berteaux N, Aptel N, Cathala G, Genton C, Coll J, Daccache A, Spruyt N, Hondermarck H, Dugimont T, Curgy JJ, Forne T, Adriaenssens E (2008). A novel H19 antisense RNA overexpressed in breast cancer contributes to paternal IGF2 expression. Mol Cell Biol.

[39] Xue Y, Gu D, Ma G, Zhu L, Hua Q, Chu H, Tong N, Chen J, Zhang Z, Wang M (2015). Genetic variants in lncRNA HOTAIR are associated with risk of colorectal cancer. Mutagenesis.

[40] Shimizu N, Nagata C, Shimizu H, Kametani M, Takeyama N, Ohnuma T, Matsushita S (2003). Height, weight, and alcohol consumption in relation to the risk of colorectal cancer in Japan: a prospective study. Br J Cancer.

[41] Cho E, Smith-Warner SA, Ritz J, van den Brandt PA, Colditz GA, Folsom AR, Freudenheim JL, Giovannucci E, Goldbohm RA, Graham S, Holmberg L, Kim DH, Malila N (2004). Alcohol intake and colorectal cancer: a pooled analysis of 8 cohort studies. Ann Intern Med.

[42] Terry P, Ekbom A, Lichtenstein P, Feychting M, Wolk A (2001). Long-term tobacco smoking and colorectal cancer in a prospective cohort study. Int J Cancer.

[43] Moran VA, Perera RJ, Khalil AM (2012). Emerging functional and mechanistic paradigms of mammalian long non-coding RNAs. Nucleic Acids Res.

[44] Halvorsen M, Martin JS, Broadaway S, Laederach A (2010). Disease-associated mutations that alter the RNA structural ensemble. PLoS Genet.

[45] Wu H, Zheng J, Deng J, Hu M, You Y, Li N, Li W, Lu J, Zhou Y (2013). A genetic polymorphism in lincRNA-uc003opf. 1 is associated with susceptibility to esophageal squamous cell carcinoma in Chinese populations. Carcinogenesis.

[46] Ma L, Zhu L, Gu D, Chu H, Tong N, Chen J, Zhang Z, Wang M (2013). A genetic variant in miR-146a modifies colorectal cancer susceptibility in a Chinese population. Arch Toxicol.

[47] Gruber AR, Lorenz R, Bernhart SH, Neubock R, Hofacker IL (2008). The Vienna RNA websuite. Nucleic Acids Res.

