# Supplementary material for: Association of genetic variants in lncRNA H19 with risk of colorectal cancer in a Chinese population
Source: Oncotarget. 2016 Mar 24;7(18):25470–7. doi: 10.18632/oncotarget.8330 (PMC5041918; doi:10.18632/oncotarget.8330)
Supplement: Supplementary file 1 [file oncotarget-07-25470-s001.pdf]

Association of genetic variants in lncRNA *H19* with risk of colorectal cancer in a Chinese population

Supplementary Materials

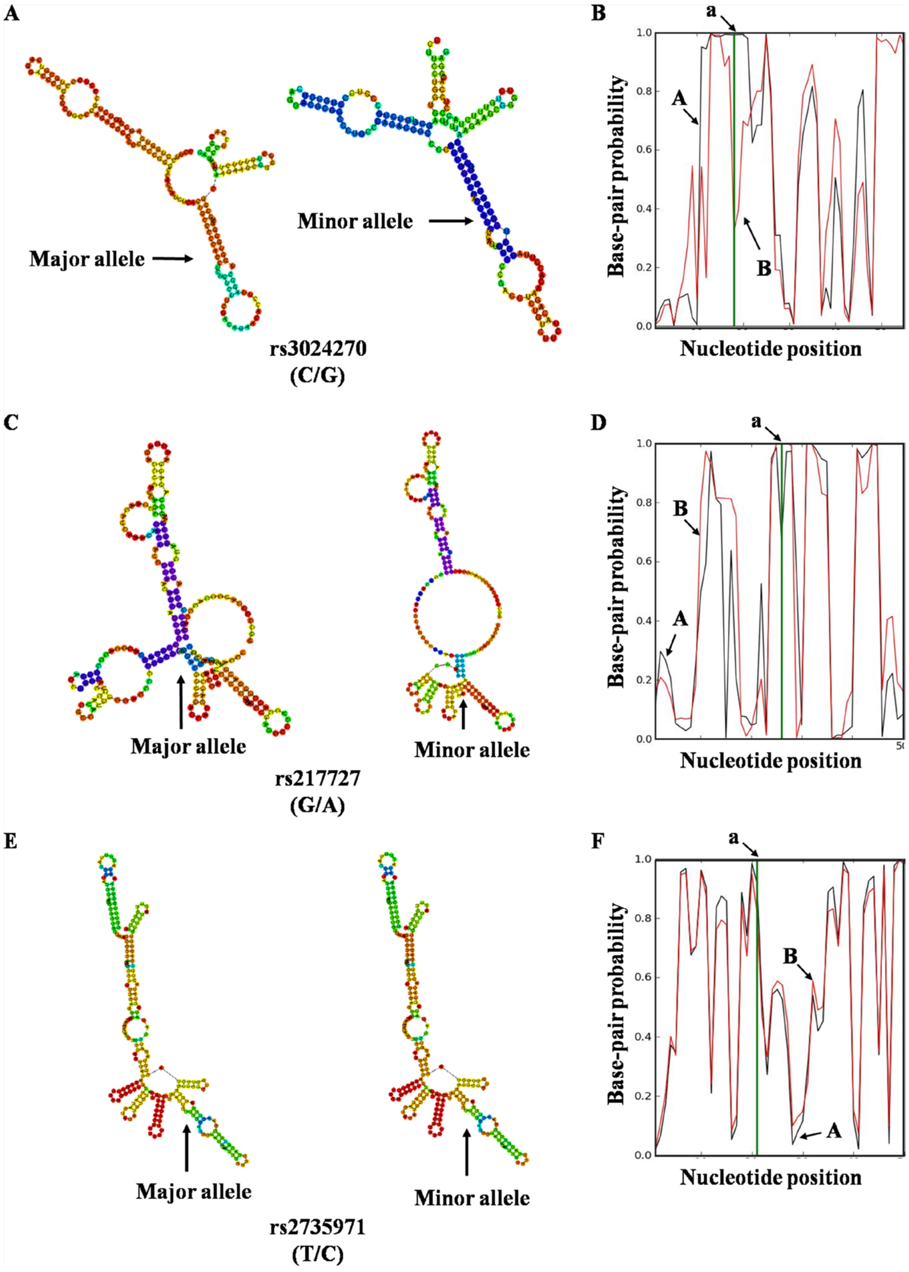

Supplementary Figure S1: Bioinformatics predictions of rs3024270, rs217727 and rs2735971 on *H19* folding structure. The folding structure alterations were demonstrated by RNAfold (**A**, **C**, **E**) and SNPfold (**B**, **D**, **F**), respectively. Arrow (Minor allele) indicates the change in structure caused by selected SNPs. Arrow a indicates the position of selected SNPs. Arrow A indicates the sequences of major allele, whereas arrow B indicates minor allele.

Supplementary Table S1: Association of tagSNPs with the risk of colorectal cancer in four genetic models

tagSNPs	Additive model		Dominant model		Recessive model		Codominant model[b]			
							het		hom	
	Adjusted OR (95% CI)[a]	*P*	Adjusted OR (95% CI)[a]	*P*	Adjusted OR (95% CI)[a]	*P*	Adjusted OR (95% CI)[a]	*P*	Adjusted OR (95% CI)[a]	*P*
rs2839698	**1.20 (1.05–1.36)**	0.007	**1.20 (1.02–1.41)**	0.030	**1.46 (1.07–1.99)**	0.017	1.14 (0.96–1.35)	0.128	**1.54 (1.12–2.12)**	0.008
rs3024270	1.11 (0.99–1.24)	0.079	1.06 (0.90–1.26)	0.482	**1.29 (1.04–1.58)**	0.019	0.99 (0.83–1.19)	0.910	**1.28 (1.01–1.61)**	0.041
rs217727	0.89 (0.79–1.00)	0.056	**0.84 (0.71–1.00)**	0.044	0.89 (0.71–1.13)	0.349	0.85 (0.71–1.01)	0.070	0.82 (0.64–1.06)	0.122
rs2735971	0.89 (0.77–1.03)	0.125	0.85 (0.72–1.01)	0.061	1.06 (0.68–1.66)	0.801	**0.83 (0.70–1.00)**	0.045	1.00 (0.64–1.57)	0.995

OR, odds ratio; CI, confidence interval.

[a]Adjusted for age, sex, smoking and drinking status in logistic regression model.

[b]het: heterozygote versus major homozygote; hom: minor homozygote versus major homozygote.

Supplementary Table S2: Subgroup analysis of clinicopathologic variables for the association between rs2839698 genotypes and colorectal cancer risk

Variables	Genotypes for rs2839698 (cases/controls)		Adjusted OR (95% CI)	P^a
	GA/AA, *n* (%)	GG, *n* (%)		
Controls (*n* = 1203)	537 (44.6)	666 (55.4)		
Cases (*n* = 1147)	564 (49.2)	583 (50.8)	**1.21 (1.02–1.43)**	0.025
Tumor site				
Colon	279 (49.9)	280 (50.1)	**1.25 (1.02–1.52)**	0.033
Rectum	285 (48.5)	303 (51.5)	1.16 (0.95–1.41)	0.147
Tumor grade				
Poor	45 (52.9)	40 (47.1)	1.38 (0.89–2.14)	0.157
Moderate	419 (47.6)	461 (52.4)	1.13 (0.95–1.34)	0.186
Well	100 (55.0)	82 (45.0)	**1.54 (1.13–2.11)**	0.007
Duke's stage				
A + B	273 (46.2)	318 (53.8)	1.06 (0.87–1.30)	0.552
C + D	291 (52.3)	265 (47.7)	**1.37 (1.12–1.68)**	0.002

OR, odds ratio; CI, confidence interval.
[a]Adjusted for age, sex, smoking and drinking status in logistic regression models.

Supplementary Table S3: Information of predicted target miRNAs gain/loss caused by rs2839689

miRNAs (loss)	Energy by miRanda	miRNAs (gain)	Energy by miRanda
hsa-miR-566	–25.65	hsa-miR-612	–29.62
hsa-miR-4486	–18.03	hsa-miR-5189	–22.36
hsa-miR-24-2-5p	–17.84	hsa-miR-1285-3p	–16.98
hsa-miR-24-1-5p	–17.68	hsa-miR-3187-5p	–20.04

Supplementary Table S4: Primers and probes for *H19* tagSNPs genotyping

TagSNPs	Primer (5′-3′)	Probe (5′-3′)
rs2839698	F: CATCGTCCCCAGCTGATGTC	G allele: FAM-CTGGGCGCCTACT-MGB
	R: GGAGTGATGACGGGTGGAG	A allele: FAM-CCTGGGCACCTAC-MGB
rs3024270	F: CTTCCTGGTGAGCGTGTCTG	C allele: FAM-TCACTGCCCCCACCT-MGB
	R: AAGTAAAGAAACAGACCCGCTTCTT	G allele: FAM-TCACTGCCCCGACCT-MGB
rs217727	F: CAAAGAGACAGAAGGATGAAAAAGAA	G allele: FAM-TCAACCGTCCGCCG-MGB
	R: CGGCGACTCCATCTTCATG	A allele: FAM-TCAACCGTCCACCGC-MGB
rs2735971	F: CACCTCCGATTCCACAACTACA	T allele: FAM-CCAATTCTGTGCCATC-MGB
	R: GAGGCTTCCCCTTCAGTCTCA	C allele: FAM-CAATTCCGTGCCATC-MGB
